# Pincho: A Modular Approach to High Quality De Novo Transcriptomics

**DOI:** 10.3390/genes12070953

**Published:** 2021-06-22

**Authors:** Randy Ortiz, Priyanka Gera, Christopher Rivera, Juan C. Santos

**Affiliations:** 1 Department of Biology, St. John’s University, Queens, NY 11439, USA; randy.ortiz16@my.stjohns.edu (R.O.); c94rivera@gmail.com (C.R.); 2 Department of Languages and Literatures, St. John’s University, Queens, NY 11439, USA; pgera2009@gmail.com

**Keywords:** NGS, RNA-sequencing, transcriptome assembly, software management, automation

## Abstract

Transcriptomic reconstructions without reference (i.e., de novo) are common for data samples derived from non-model biological systems. These assemblies involve massive parallel short read sequence reconstructions from experiments, but they usually employ ad-hoc bioinformatic workflows that exhibit limited standardization and customization. The increasing number of transcriptome assembly software continues to provide little room for standardization which is exacerbated by the lack of studies on modularity that compare the effects of assembler synergy. We developed a customizable management workflow for de novo transcriptomics that includes modular units for short read cleaning, assembly, validation, annotation, and expression analysis by connecting twenty-five individual bioinformatic tools. With our software tool, we were able to compare the assessment scores based on 129 distinct single-, bi- and tri-assembler combinations with diverse k-mer size selections. Our results demonstrate a drastic increase in the quality of transcriptome assemblies with bi- and tri- assembler combinations. We aim for our software to improve de novo transcriptome reconstructions for the ever-growing landscape of RNA-seq data derived from non-model systems. We offer guidance to ensure the most complete transcriptomic reconstructions via the inclusion of modular multi-assembly software controlled from a single master console.

## 1. Introduction

Homemade de novo transcriptomic workflows tend to be idiosyncratic to specific study goals, unoptimizable to other studies and, in many cases, left unpublished or buried in supplementary materials. We could say Rnnotator [1] in 2010 was the first single-assembler transcriptomic pipeline to be publicly available, while the Oyster River Protocol (ORP; [2]) in 2018 was the first multi-assembler pipeline available. This presumed eight-year period between single- and multi-assembler approaches is odd considering multi-assembler methods have been shown to produce reconstructions with higher degrees of completeness [2]. Nevertheless, the combinations of assemblers that produce the best reconstructions in the multi-assembly approach are not well explored nor classified. Adding to the complexity of the situation, assemblers are routinely updated, and new assemblers are created in a timely fashion, making assembler comparisons both a necessity and routine process. The closest comparison to our workflow would be the ORP; however, it employs a rigid tri-assembly approach to produce high quality transcriptomes via rnaSPAdes (k55, k75; [3]), Trinity (k25; [4]) and Shannon (k75; [2,5]). In comparison, we developed an open-source workflow that broadens the k-mers used to up to five total k-mers per assembler. Our software, Pincho [6], allows the user to design and customize their own k-mer list and number of assemblers, among other parameters.

To characterize our management software, we present two major goals of this study that we sought to complete. The first was to construct a publicly available and customizable modular management toolkit that could simplify de novo transcriptomic work for data scientists. This simplification took place via the amalgamation of well-established and reviewed genomic and transcriptomic software centralized in one quick download and even faster user implementation options. We customize this workflow with the most common software used in de novo transcriptomics along with the modularity to allow simple incorporation of new software as future tools become available. Our second goal is to provide a comprehensive analysis on de novo transcriptome assembler performance individually and in combination. To our knowledge, this is the first publication on synergistic effects of single-, bi-, and tri-assembly combinations between nine distinct de novo and reference-guided assemblers aimed to elevate de novo transcriptome quality and completeness.

## 2. Materials and Methods

### 2.1. Components of the Pincho Workflow

**Figure 1:**
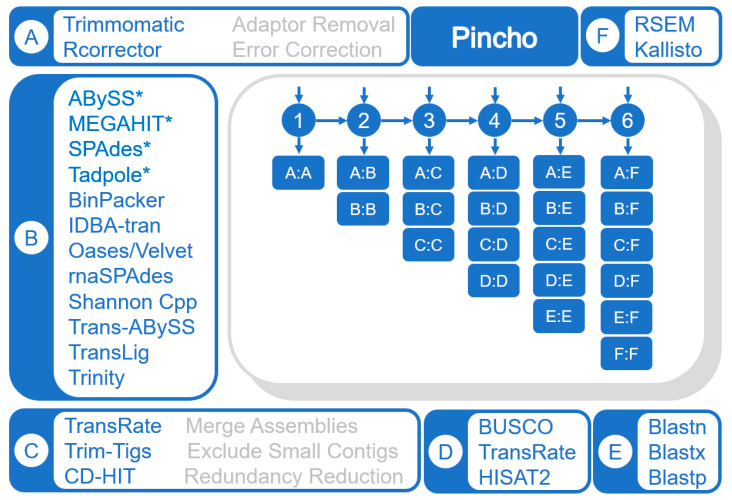
Pincho Management Workflow. Software installed in the Pincho workflow v0.1, including (**A**) pre-processing, (**B**) transcriptome and * genome assemblers, (**C**) post-processing, (**D**) assessment software, (**E**) annotation software, and (**F**) expression analysis software. Modules may begin at any position (**A**–**F**) but must then process sequentially (i.e., **B**, **C**, **D**…). Possible avenues depicted in shorthand, where A:D represents steps **A**, **B**, **C** and **D**. Any number of items may be called from each module (i.e., module B: IDBA-tran, Trans-ABySS, Trinity = 3 items called from module B).

Our software supports various applications and automates their parameter, computer resources and output management via Python3 and Bash (Supplementary Figure S1). Pincho consists of twenty-five functions which fall under six modules: preprocessing (adaptor removal with Trimmomatic [7,8] and error correction via Rcorrector [9,10]); de novo assembly (ABySS [11,12], Tadpole [13,14], BinPacker [15,16], IDBA-tran [17,18], MEGAHIT [19,20], Oases/Velvet [21,22], rnaSPAdes [3,23], Shannon Cpp [5,24], SPAdes [25,26], Trans-ABySS [27,28], TransLig [29,30], and Trinity [4,31], Table 1; post-assembly (consensus assembly generation with TransRate [32,33], isolation of short transcripts under bp length threshold and redundancy reduction via CD-HIT [34,35]); assembly assessment (alignments to reference transcriptomes or to the original raw reads via HISAT2 [36,37], BUSCO [38,39] and TransRate); annotation using a user reference (NCBI BLASTX, BLASTN, and BLASTP; [40,41,42]); and expression analysis (kallisto [43,44] and RSEM [45,46], Figure 1 and Supplementary Figure S1). Several important notes: Pincho can process Sequence Read Archive (SRA, [47]) data accession numbers via SRAtoolkit [48], Trinity can be run in genome guided mode instead of *De novo* with help from Samtools [49,50], and TransLig was modified to include assembly lengths via SeqKit [51,52].

### 2.2. Dataset Criteria and Selection

We analyzed eight distinct non-model datasets from the SRA ([53]; Table 2. We focused on hyloid anurans (frogs) that have complex and usually large genomes (e.g., ~6.76 Gb for *Dendrobates pumilio*, [54]). Data was chosen via the following criteria: (a) publicly sourced RNA-seq data, (b) paired-end reads of various insert sizes (Table 2), (c) fastq format, (d) Illumina sequencing, (e) non-model organisms, (f) data containing a base count lower than 2Gb and (g) data that passed Pincho’s rapid assessment with a complete BUSCO score greater than 50%. Rapid assessment is composed of fasterq-dump download of SRR raw reads, removal of Illumina adaptors, if necessary, from raw data via Trimmomatic, assembly of reads via succinct *de Bruijn* graphs with MEGAHIT and assessment via BUSCO scores. Chosen SRA files were analyzed with FastQC [55], revealing that all files were adapter free.

Our datasets are purposely under the standard yield of RNA-seq experiments (2GB –4GB), to highlight the potential of the selected assemblers on low yield, low coverage datasets. As higher levels of sequencing coverage lead to higher quality NGS data [56], we chose NGS data that are most likely to contain low sample coverage owing to low read counts [57]. We selected smaller sized files on average 6.88M reads, which is well beneath the recommended sequencing read number of 20M [56] to ensure an NGS scenario of low coverage. As a balance we made sure that all files were at least above 50% in complete BUSCO scores to avoid scenarios where read coverage was insufficient. Low coverage datasets are prone to many types of assembly errors (i.e., fragmentation and incompleteness [32]), which allows us to accurately test the various types of algorithms employed by the tested transcriptome assemblers and their abilities to work with problematic datasets. It is only under this scope that we can ideally view assembler performance and synergy without the reliance on synthetic data. We expect that if assemblers succeed at reconstructing more from smaller datasets, then they are sensitive enough to use on larger datasets as well.

### 2.3. Pincho Workflow Implementation

Raw data was analyzed with the Pincho pipeline with the following configurations: SRA accession numbers were used to download data from the SRA database via fasterq-dump followed by whitespace removal and compression. Leading and lagging low quality base removal was performed via Trimmomatic, followed by error correction by Rcorrector. Transcriptomes were assembled via Trans-ABySS, BinPacker, IDBA-tran, Shannon Cpp, rnaSPAdes, TransLig, Trinity, MEGAHIT (positive control) and Tadpole (negative control) with adaptive k-mer control enabled. Adaptive k-mer control utilizes a minimum k-mer of k21 and four k-mers generated based on their respective maximum insert length and middle three quartiles between k21 and the maximum. Consensus assembly generation was conducted via TransRate. Read mapping was performed via HISAT2 aligner, presence of ancestral genes was identified by BUSCO and n50/n90 were calculated via TransRate. Assessment was conducted in combinations between the nine assemblers individually and in groups of two and three. Oases was not utilized in this study due to the frequent unresolved bugs associated with the software and its lack of maintenance (last major update 20 May 2013). SPAdes and ABySS de novo genome assemblers were not utilized in this study as we used their transcriptomic counterparts designed for transcriptome assembly. Both rnaSPAdes and ABySS were demonstrated to outperform SPAdes and ABySS, respectively [3,27].

### 2.4. K-mer Size Determination

K-mer sizes were left to their default values (Table 1) if the assembler only allowed one k-mer size as input and assembler runtime was extensive. Therefore, default k-mers were used for BinPacker, TransLig, Trinity and Shannon Cpp. Assemblers that allowed the selection of multiple k-mer sizes and/or were time efficient were assigned a broad range of five k-mer sizes.

### 2.5. Assessment Validation

We utilized three metrics (TransRate, BUSCO, and HISAT2) that best represent the quality of a de novo transcriptome. TransRate provides the n50/n90 statistic, among others, which is the largest contig size where 50%/90% of bases are contained in transcripts of this length. These n50/n90 scores are often used to ascertain the quality of a reconstruction, with longer n50/n90 lengths correlating to a more complete assembly. Other assessment metrics include complete BUSCO scores representing percent ancestral transcripts present and HISAT2′s overall alignment score which is the percentage of raw data utilized within reconstructions. For our workflow, we used BUSCO’s Eukaryota dataset as a reference.

Respective assessment scores were judged per assembler as greater than MEGAHIT’s assessment scores or less than MEGAHIT’s assessment scores. Assessment scores (AS) greater than MEGAHIT were subjected to the following formula:

(1) $$\frac{AS_{X}}{AS_{MAX}}\times 0.5$$

AS less than MEGAHIT were processed under a different formula to calculate underperformance:

(2) $$\frac{AS_{X}}{-AS_{MIN}}\times 0.5$$

while scores equal to MEGAHIT were counted as 0. Average assessment scores (AAS) were calculated as the average of HISAT2′s overall alignment, complete BUSCO score, and TransRate’s n50/n90 scores in a 1:1:1 ratio, so n50 and n90 scores were averaged together before averaging with the other two assessment scores. Finally, the AAS were normalized between the numbers of 0.5 as overperforming versus MEGAHIT and −0.5 as underperforming.

## 3. Results

### 3.1. Workflow Installation, System Build, and Performance

Pincho is packaged with an installer script written in Python3 and Bash which will install and configure required dependencies in Linux Ubuntu systems. Our workflow requires a minimum of 24 threads and 128GB of memory to run efficiently and is largely GPU independent. It is recommended to scale performance parameters evenly if higher performance is desired (i.e., 24:128 ratio). Our study was conducted on two new workstations including: AMD Ryzen 9 3900X 3.8GHz processor, G.Skill 128GB 4 × 32 D4 3200 memory modules, and an ASUS TUF GAMING X570-PLUS motherboard. An alternative replica build would be to purchase a PowerSpec G464 and upgrade the memory modules to a total of 128GB (net price 2200 USD). Our test data ranged in both number of bases and file size (Table 1) to provide an accurate depiction of the capacities of our workflow performance. We encountered no errors conducting the study with the parameters stated above. Methods can be easily replicated via Pincho’s completely modular user interface.

### 3.2. Average Assessment Score Generation

We utilize three distinct assessment software––HISAT2, TransRate, and BUSCO––to derive raw scores for each single-, bi-, and tri- assembly run (see assessment validation in methods) and mark their over/underperformance in regard to a MEGAHIT single assembly run. Individual metric scores are normalized to a scale between −0.5 and 0.5, where 0 is equal to a MEGAHIT single assembly run assessment score. Negative integers denote underperformance and positive integers denote overperformance when compared to MEGAHIT genome assembler. Individual assessment scores are then averaged together respectively to provide an AAS per assembler or assembler group. The following assemblers were utilized in this study: Trans-ABySS, BinPacker, IDBA-tran, Shannon Cpp, rnaSPAdes, TransLig, Trinity, MEGAHIT, and Tadpole.

### 3.3. Single-Assembly

**Figure 2:**
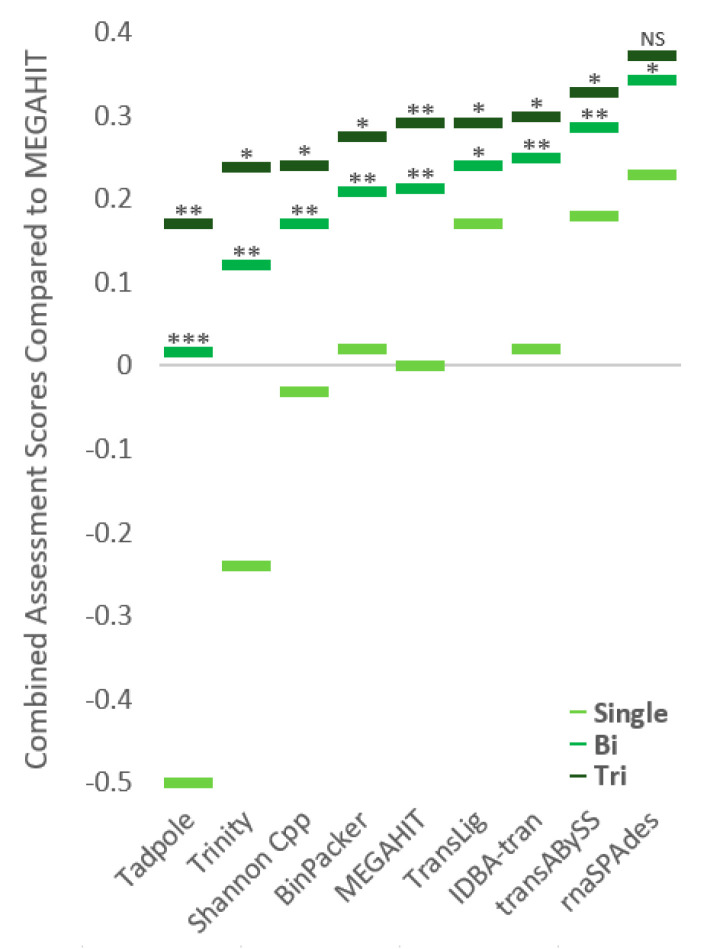
Single-, bi-, and tri-assembly assessment score averages. Average assessment scores from single-, bi-, and tri-assembly runs compared to MEGAHIT single-assembly as a baseline score (0). Scores lower than 0 underperformed when compared to MEGAHIT single-assembly, whereas, scores higher than 0 overperformed. Average assessment scores calculated by the average of HISAT2 overall alignment, BUSCO complete score, and TransRate n50 and n90 metrics averaged across all files processed. Assemblers utilized are included in the x-axis to denote both their average scores for single assembly and their average scores as part of a pair of two or three. Two tailed paired T-tests were conducted between single-assembly and bi-assembly, and between bi-assembly and tri-assembly. P-values are noted between single- and bi-assembly combinations and between bi- and tri-assembly combinations. All comparisons conform to *p* < 0.05 except for no-significance noted between bi- and tri-assembly associated with rnaSPAdes. *** is *p* < 0.00001, ** is *p* < 0.001, * is *p* < 0.05, and NS (No Significance) is *p* > 0.5. *p*-values are under FDR (False Discovery Rate) correction.

**Figure 3:**
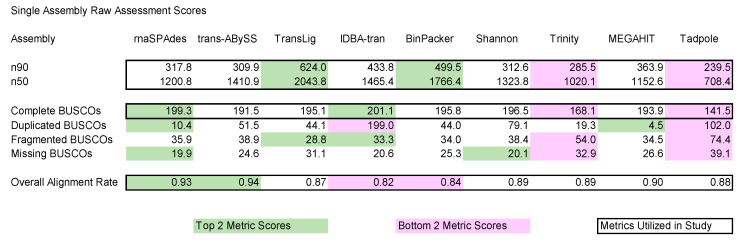
Single-assembly raw average assessment scores. Assessment metrics used in study: n50/n90 (via TransRate), complete BUSCOs (via BUSCO), and overall alignment rate (via HISAT2) are boxed in. Top two metric scores per assessment criteria are highlighted in green. Bottom two metric scores are highlighted in pink. Metrics not boxed in were provided to aid discussion but not for the generation of the average assessment scores.

According to our combination of assessment software criteria, rnaSPAdes outperformed all other assemblers with an AAS of 0.23, followed by Trans-ABySS (AAS: 0.18), TransLig (AAS: 0.17), IDBA-tran (AAS: 0.02), BinPacker (AAS: 0.02; Figure 2), and the MEGAHIT single-assembly baseline (AAS: 0). Shannon Cpp (AAS: −0.03), Trinity (AAS: −0.24), and Tadpole (AAS: −0.50) underperformed relative to the baseline (Figure 2). Runtime analysis highlights no correlation between total time consumption and performance, as assemblers that required the most time did not produce the best assemblies nor vice versa (Supplementary Figure S2). Assessment of raw data from our assessment software reveals rnaSPAdes and Trans-ABySS obtained the highest HISAT2 scores (>92%), rnaSPAdes and IDBA-tran scored the highest complete BUSCO scores (>199 complete eukaryotic ancestral transcripts), and TransLig and BinPacker contained the longest n50/n90 lengths (>1766 bp/>499 bp; Figure 3). Alternatively, IDBA-tran and BinPacker obtained the lowest HISAT2 scores (<85%), Trinity and Tadpole scored the lowest complete BUSCO scores (<169 complete transcripts) and also the shortest n50/n90 lengths (<1021 bp/<286 bp; Figure 3).

### 3.4. Bi-Assembly

**Figure 4:**
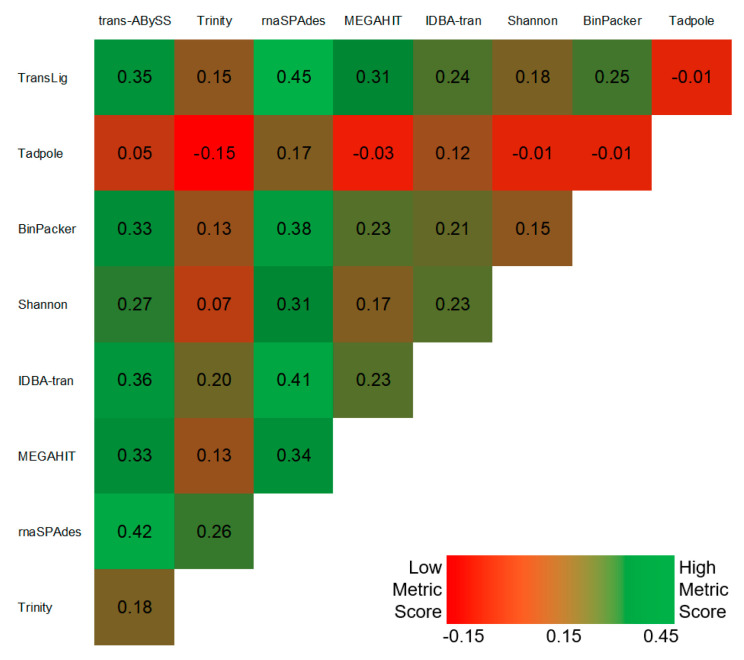
Bi-assembly assessment scores. Heatmap of bi-assembly assessment scores from 36 combinations of 9 assemblers compared to MEGAHIT single-assembly as a baseline score (0). Scores lower than 0 underperformed when compared to MEGAHIT single-assembly, whereas, scores higher than 0 overperformed. Green denotes a higher assessment score and red denotes a lower assessment score among the 36 bi-assembly groups. Shannon denotes Shannon Cpp version.

The pairing of assemblers often increased the AAS; however, our negative control Tadpole caused a decrease in metric scores of our previous top three single-assemblers: rnaSPAdes (Net ∆AAS: −0.06), Trans-ABySS (Net ∆AAS: −0.13), and TransLig (Net ∆AAS: −0.18; Figure 4). The combination of TransLig and rnaSPAdes outperformed all other single- and bi-assembly combinations achieving an AAS of 0.45 (Figure 2 and Figure 4). Pairings between Trans-ABySS and rnaSPAdes achieved the second highest AAS of 0.42 (Figure 4). Bi-assemblies involving combinations between Tadpole and either Trinity, MEGAHIT, Shannon Cpp, Binpacker, or TransLig all underperformed when compared to a MEGAHIT single-assembly run (Figure 4).

### 3.5. Tri-Assembly

**Figure 5:**
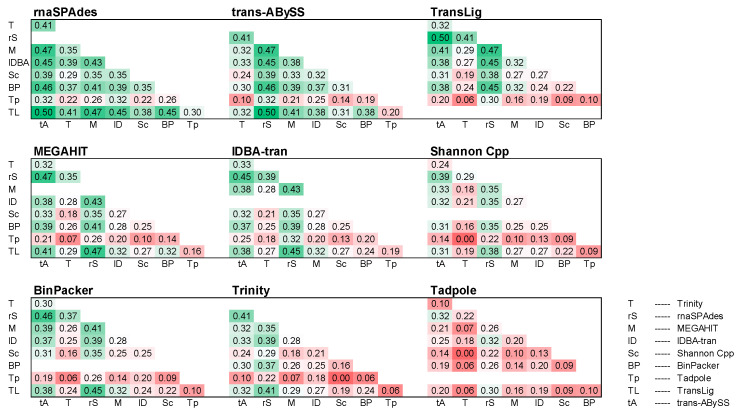
Tri-assembly Scores. Tri-assembly assessment score results from 84 combinations of 9 assemblers, respectively. All assembler metrics are compared to over/underperformance to the average MEGAHIT single-assembly score. Highlighted values range from high average assembly scores up to 0.5 (green) to low average assessment scores down to 0.0 (red). Metric scores are consistent, with previous figures allowing for cross comparison.

**Figure 6:**
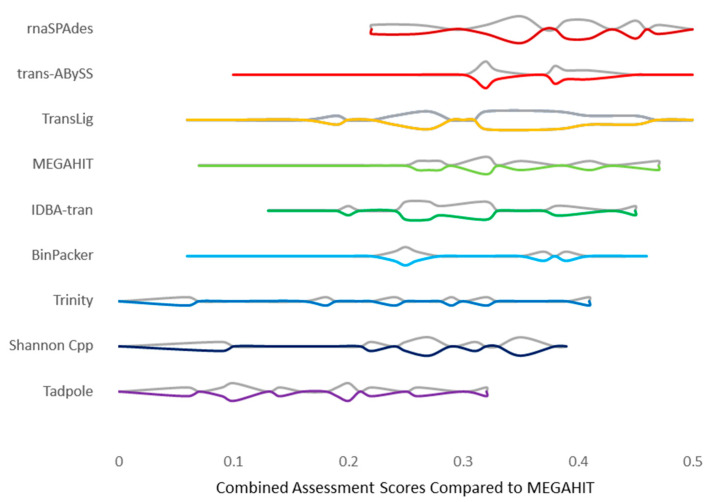
Tri-assembly score distributions. Violin plots representing assessment score frequency and distribution among tri-assembly runs. All assembler metrics are compared to over/underperformance to the average MEGAHIT single-assembly score (0). All tri-assembly scores performed equal to or greater than the baseline. Higher quality assembler combinations are represented via higher numerical scores up to a maximum of 0.5.

We observed the highest possible AAS of 0.50 in a tri-assembly approach containing Trans-ABySS, rnaSPAdes and TransLig (Figure 5). The higher AAS values are primarily located in the highest performing assembler groups: Trans-ABySS, rnaSPAdes, and TransLig (Figure 5). The lower AAS values are found not only in the negative control Tadpole, but in Trinity and Shannon Cpp as well. The rnaSPAdes bracket performed the best, yielding the highest AAS, while the Tadpole bracket performed the lowest, yielding the lowest AAS on average (Figure 5). The rnaSPAdes bracket also exhibited a smaller distribution of AAS, spanning 0.22 to 0.50, with a higher frequency of high AAS than other assembler groups (Figure 6). When tri-assembly runs are sorted from lowest AAS to highest, the rnaSPAdes group continues to lead the other tri-assembly groups at every datapoint (Supplementary Figure S3). Signs of over/underperformance amongst tri-assembly runs were observed, with Tadpole, Trinity, and Shannon Cpp tri-assembly approach underperforming by scoring equal to the MEGAHIT baseline previously set at 0 (Figure 5).

## 4. Discussion

### 4.1. Single-Assembly Mode

Single-assembler comparisons reared interesting results regarding the efficiency of de novo transcriptome assemblers compared to their genomic counterpart. Genome assemblers are known for their conservative style for reconstructions, whereas transcriptome assemblers take risks to assemble every transcript isoform identified. It is largely due to this deviation between the two software that we see gains or losses in average assessment scores. To further elaborate, isoforms are more common among longer transcripts as there is more genomic material, increasing the probability of the accumulation of mutations and change over time. As longer transcript isoforms are added to the assembly, the n50/n90 lengths increase. It also helps generate more ancestral transcripts, as each variant has a chance to align to the reference eukaryotic database of ancestral transcripts. Finally, more raw data containing variant fragments will be incorporated into the product, resulting in a higher HISAT2 alignment score. In summary, de novo transcriptome assemblers should be more than capable of outperforming de novo genome assemblers in part due to the identification and reconstruction of isoforms, which is why it is so bizarre to observe some transcriptome assemblers unable to outperform genome assemblers (i.e., MEGAHIT).

#### 4.1.1. Trinity

Perhaps the most perplexing of all our results was the tendency for Trinity to underperform, as it has long been described in literature to be quite robust at de novo transcriptome assembly. Trinity incorporated roughly 89% of raw data into its assembly, which is average among assemblers tested (Figure 3). Trinity’s n50/n90 scores, however, were roughly half of what TransLig, a non-adaptive k-mer assembler, produced. The short n50/n90 lead us to believe that Trinity may be unable to bridge fragmented transcripts as well as other assemblers. Upon examination of the fragmented BUSCO scores, we observe that Trinity in fact did fragment more transcripts than other assemblers.

#### 4.1.2. Shannon Cpp

Reconstructions produced by Shannon Cpp were fairly close to MEGAHIT’s AAS. Shannon Cpp exhibited a higher n50 score than MEGAHIT, but a lower n90 score. Shannon Cpp tended to fragment more ancestral genes than MEGAHIT and that higher rate of fragmentation may account for the lower n90 score. Shannon Cpp had a higher complete BUSCO score than MEGAHIT; however, Shannon Cpp utilized roughly 1% less of the raw NGS datasets than MEGAHIT did, leading to a lower average assessment score total. Shannon Cpp utilizes a type of information theory algorithm built on a de Bruijn graph and we speculate that this algorithm is more conservative than MEGAHITs more normalized de Bruijn graph method, leading to more fragments and less raw NGS integration.

#### 4.1.3. Tadpole and MEGAHIT

Tadpole, as a basic assembly tool, is not as complex as the other assemblers and tends to create many problematic reconstructions (i.e., chimeras, nonsense repeat sequences, etc.). This is evident as Tadpole scored the lowest in every assessment metric, except for raw NGS data incorporation. Further exploration of Tadpole assemblies reveals obvious mis-assemblies. This was known before the study and is why we chose Tadpole as a negative control: a metric to use as an indication of poor assembly methodology. In addition, we have included what we perceive to be the best genome assembler, MEGAHIT (as a single assembler), to act as a baseline for transcriptome assemblies. Genome assemblers tend to be more conservative with their reconstructions and therefore will score moderately well according to assessment software metrics; however, they do not account for isoforms which account for large portions of transcriptomes. This allows for transcriptome assemblers to elevate themselves from the MEGAHIT baseline by providing isoform assemblies that are of high quality to increase their metric scores higher than that of a genome assembler.

#### 4.1.4. BinPacker

With the second highest n50/n90 scores and decent complete BUSCOs, BinPacker ranks among the top assemblers, but on average, BinPacker’s performance is only slightly better than MEGAHIT’s. BinPacker was poor at integrating the raw NGS data into the completed reconstruction, scoring among the bottom two in the HISAT2 assessment bracket. Data integration depends on the quality of the raw reads, but also whether the algorithm designed for the assembler was able to incorporate that read within the assembly. BinPacker underperformed in raw NGS data incorporation; however, TransLig, the sequel to BinPacker, improves on this flaw.

#### 4.1.5. IDBA-Tran

Suffering from the same issue as BinPacker, IDBA-tran’s low raw NGS data utilization rate detracts from its impressive complete BUSCO score and decent n50/n90 metrics. Fortunately, its two strengths can carry IDBA-tran over the MEGAHIT baseline, providing evidence that IDBA-tran provides reconstructions of better quality than a genome assembler. An oddity is IDBA-tran’s tendency to duplicate BUSCOs, which may be caused by the addition of five k-mer sizes and the inability for IDBA-tran to reduce redundancy among the assembly.

#### 4.1.6. TranLig, Trans-ABySS, and rnaSPAdes

Top three de novo assemblers are rnaSPAdes, Trans-ABySS, and TransLig. In single-assembly comparisons these de novo transcriptome assemblers were able to largely outperform the other assemblers in various assessment metrics. Complete BUSCOs and raw data utilization rates for rnaSPAdes were both part of the top two metric scores, so it is no surprise rnaSPAdes scored the best among the three. rnaSPAdes was also able to produce one of the least redundant assemblies. Trans-ABySS incorporated the most NGS data into its assembly procedure but was not able to reconstruct as many transcripts as rnaSPAdes nor TransLig. TransLig outperformed all assemblers in n50/n90 scores, however its raw NGS data utilization was lacking. It is clear from the investigated assessment metrics that each of these assemblers excel in one area or another, which is precisely why multi-assembly provides higher quality transcriptomes.

### 4.2. Bi-Assembly Mode

Bi-assembly methods, including Tadpole, led to lower overall assessment scores when compared to pairings without Tadpole, cementing the validity of our negative control. On average, all pairings excluding Tadpole achieved scores greater than the MEGAHIT baseline and greater AAS when compared to the single assembly approach. Bi-assembly increased n50/90 scores, complete BUSCOs and overall alignment scores from their single assembly counterparts on average. All increases are expected as we nearly double the coverage, while including several transcripts that were missed in the single assembler methodology. We note a significant increase in AAS from single- to bi-assembly approaches across all assemblers. Lastly, we note rnaSPAdes produced the top three bi-assembly reconstructions, providing further evidence of the positive synergistic effects of our top single-assemblers.

### 4.3. Multi-Assembly Mode

We have demonstrated the potential of utilizing the multi-assembler approach to elevate the overall quality of reconstructions across three distinct assessment criteria. We highly recommend the usage of Trans-ABySS, rnaSPAdes, and TransLig in combination for de novo transcriptome assembly as they provided the highest metric scores. We observe the highest single-assembly AAS at 0.23, highest bi-assembly at 0.45 and the highest tri-assembly at the maximum 0.50 demonstrating that assemblers can not only synergize well together, but also that bi-assembly increased the quality of single-assembly by a large margin. We observe a significant increase in scores from bi- to tri-assembly as well. Although rnaSPAdes observed no significant change in average scores, there was still an increase in the number of novel transcripts recorded (via BUSCO) and this metric alone is worth the addition of a third assembler. We advocate for the usage of multi-assembler workflows as they provide the best chances of complete assemblies for non-model organisms.

## 5. Conclusions

Over the past ten years, researchers have provided us with an extensive coverage of the strengths and weaknesses of the various de novo transcriptome assemblers in single-assembly approaches. However, there have been scarce publications to date offering a comprehensive comparison between multi-assembly approaches. We offer a broad comparative review of seven well-maintained de novo transcriptome assemblers and two de novo genome assemblers scored via three distinct assessment criteria. All our work was completed via a modular pipeline, Pincho, which we contribute to the scientific community as a modern modular de novo transcriptomic workflow written in Python3 for Ubuntu 20.04 Focal Fossa LTS on our GitHub page.

## Figures and Tables

**Table 1:** De novo Assemblers Utilized in Pincho.

Assembler	Genome or Transcriptome	K-mer Used	K-mer Default	Algorithm	Version	Version Release	Software Release	Cited by ^2^	Datasets Explored
ABySS	Genome	Adaptive	32	*de Bruijn* Graph	v2.2.4	1/30/2020	11/26/2008	3481	Human
BinPacker	Transcriptome	25	25	Splice Graph	v1.0	10/17/2019	3/19/2015	95	Human, Mouse, Dog
IDBA-tran	Transcriptome	Adaptive	20, 30, 40, 50	*de Bruijn* Graph	v1.1.3	6/11/2016	6/19/2013	155	*Oryza sativa*
MEGAHIT	Genome	Adaptive	21, 41, 61, 81, 99	*de Bruijn* Graph	v1.2.9	10/14/2019	9/25/2014	1738	Soil
Oases/Velvet	Transcriptome	Adaptive	19, 21, 27, 31, 35	*de Bruijn* Graph	v0.2.08/ v1.2.10	05-20-2013/10-17-2013	12-11-2011/11-16-2007	1437	Human, Mouse
rnaSPAdes	Transcriptome	Adaptive	Automated k-mers	*de Bruijn* Graph	v3.14.1	5/2/2020	11/16/2018	122	Humans, Mouse, Corn, *Arabidopsis*
Shannon Cpp	Transcriptome	25	25	*de Bruijn* Graph	v0.4.0	12/19/2019	2/9/2016	27	Human
SPAdes	Genome	Adaptive	21, 33, 55	*de Bruijn* Graph	v3.14.1	5/2/2020	5/7/2012	12635	*Escherichia coli, Deltaproteobacteria*
Tadpole	Genome	Adaptive	31	Simple Kmer Code	v38.86	6/13/2020	1/9/2012	437	Fungus, Bacteria, Plant, Soil
Trans-ABySS	Transcriptome	Adaptive	32	*de Bruijn* Graph	v2.0.1	2/19/2018	6/18/2010	467	Human
TransLig	Transcriptome	31	31	Line Graph Iterations	v1.3	10/26/2019	11/23/2018	7	Human, Mouse
Trinity ^1^	Transcriptome	25	25	*de Bruijn* Graph	v2.11.0	6/30/2020	12/3/2010	1175	*Drosophila melanogaster*

^1^ genome guided mode available. ^2^ cited by column updated on 15 June 2021.

**Table 2:** Test NGS Dataset from NCBI SRA database.

Species	Accession	BUSCOs (%) ^1^	Reads (M)	Bases (G)	Read Length (bp)	File Size (Mb)	Tissue
*Allobates femoralis*	SRR8288062	62.4	3.5	0.8	120	504.4	Skin
*Amazophrynella minuta*	SRR8288029	70.6	4.4	1.1	120	641.6	Skin
*Dendrobates auratus*	ERR3155280	91.0	3.3	1.9	294	1000.0	Skin
*Dendrobates imitator*	ERR3169394	66.3	16.3	1.6	50	782.5	Skin
*Dendrobates sirensis*	SRR8288043	72.2	4.9	1.2	120	710.7	Skin
*Lithobates catesbeianus*	SRR4048903	77.6	6.8	1.3	99	558.0	OB ^2^
*Pyxicephalus adspersus*	SRR6890710	87.8	10.0	1.5	75	538.8	Testis
*Scinax ruber*	SRR8288044	73.7	5.8	1.4	120	840.1	Skin

^1^ Complete BUSCO using Pincho’s rapid assessment at default settings ^2^ Olfactory Bulb.

## Data Availability

The data sets supporting the results of this article are available in the NCBI SRA database, under the accession numbers: SRR8288062, SRR8288029, ERR3155280, ERR3169394, SRR8288043, SRR4048903, SRR6890710, and SRR8288044.

## Supplementary Materials

The following are available online at https://www.mdpi.com/article/10.3390/genes12070953/s1, Figure S1: Pincho User Interface, Figure S2: Single-assembly runtimes, Figure S3: Tri-assembly runs sorted by lowest to highest assessment scores are each uploaded separately from main text.
