# Supplementary material for: Pincho: A Modular Approach to High Quality De Novo Transcriptomics
Source: Genes (Basel). 2021 Jun 22;12(7):953. doi: 10.3390/genes12070953 (PMC8304035; doi:10.3390/genes12070953)
Supplement: Supplementary file 1 [file genes-12-00953-s001.zip › Supplemental_Figures S1-S3.pdf]

genes
MDPI

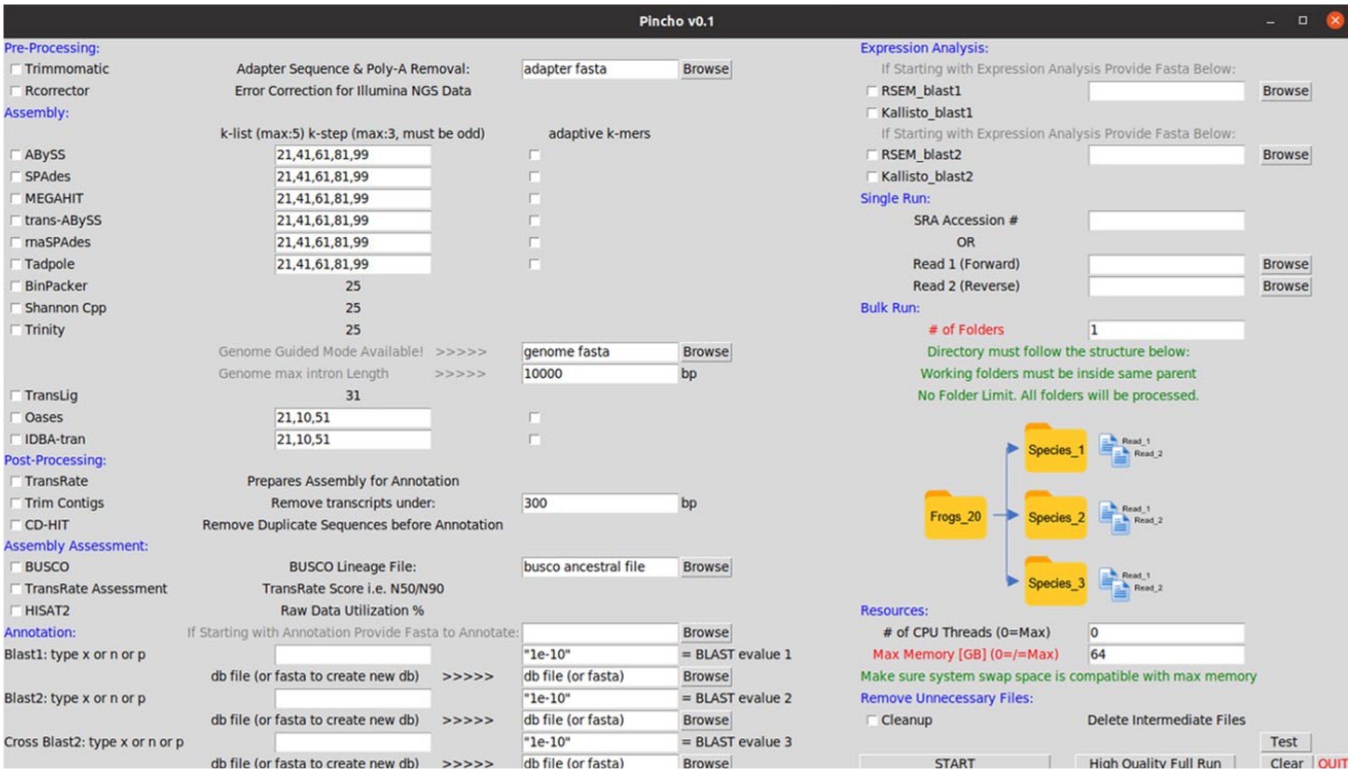

Figure S1. Pincho User Interface. Pincho v0.1 UI written in Python-TK for de novo transcriptome analysis. User can elicit as many or as few software options as necessary. Any adapter sequence can be provided for pre-processing adapter removal. K-mers may be explicitly stated or left to our software to automatically generate 5 distinct k-mers based on maximum insert sizes of the files via the adaptive k-mer option. Short transcripts may be removed from the assembly before annotation if preferred. Duplicate sequences can also be removed before annotation. Blast modes supported are blastx, blastn and blastp. Databases can be automatically created by Pincho if a fasta is provided. We offer the ability to annotate blast results against a second database if necessary. Pincho capable of working from SRA accessions, local reads from the user, and even able to process data in bulk without the need to manually reassign the workflow. We offer the option to remove intermediate files to conserve computer space.

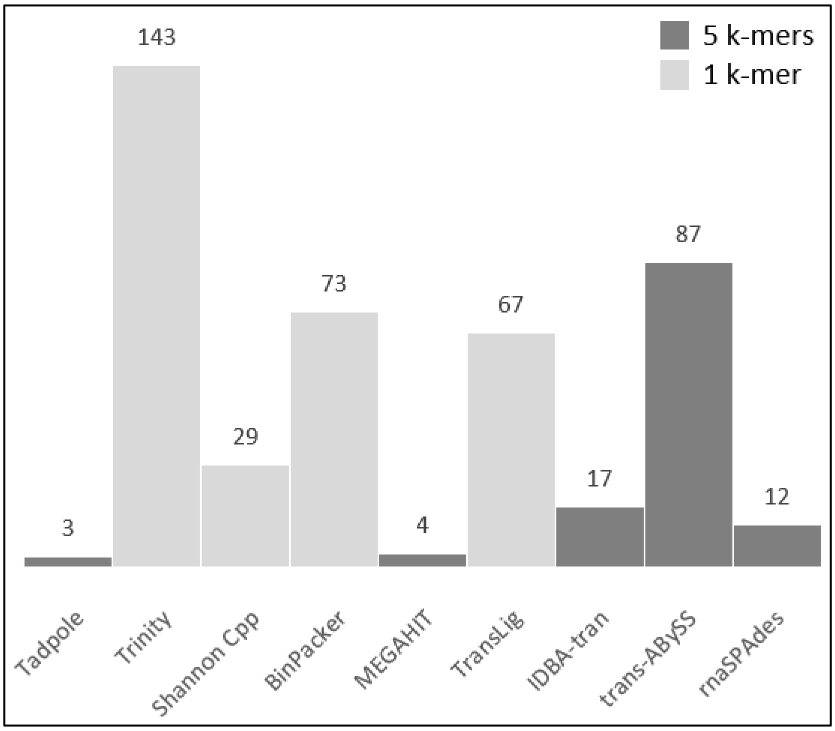

Figure S2. Single-assembly runtimes. Average runtimes for each assembler in minutes. Assemblers were run with 24 threads (Ryzen 9 3900X AMD chipset), 120GB of memory (G.Skill 128GB 4x32 D4 3200 memory modules) if allowed. Dark grey bars denote the usage of five k-mers created by Pincho's adaptive k-mer option. Light grey bars denote the usage of default k-mer settings.

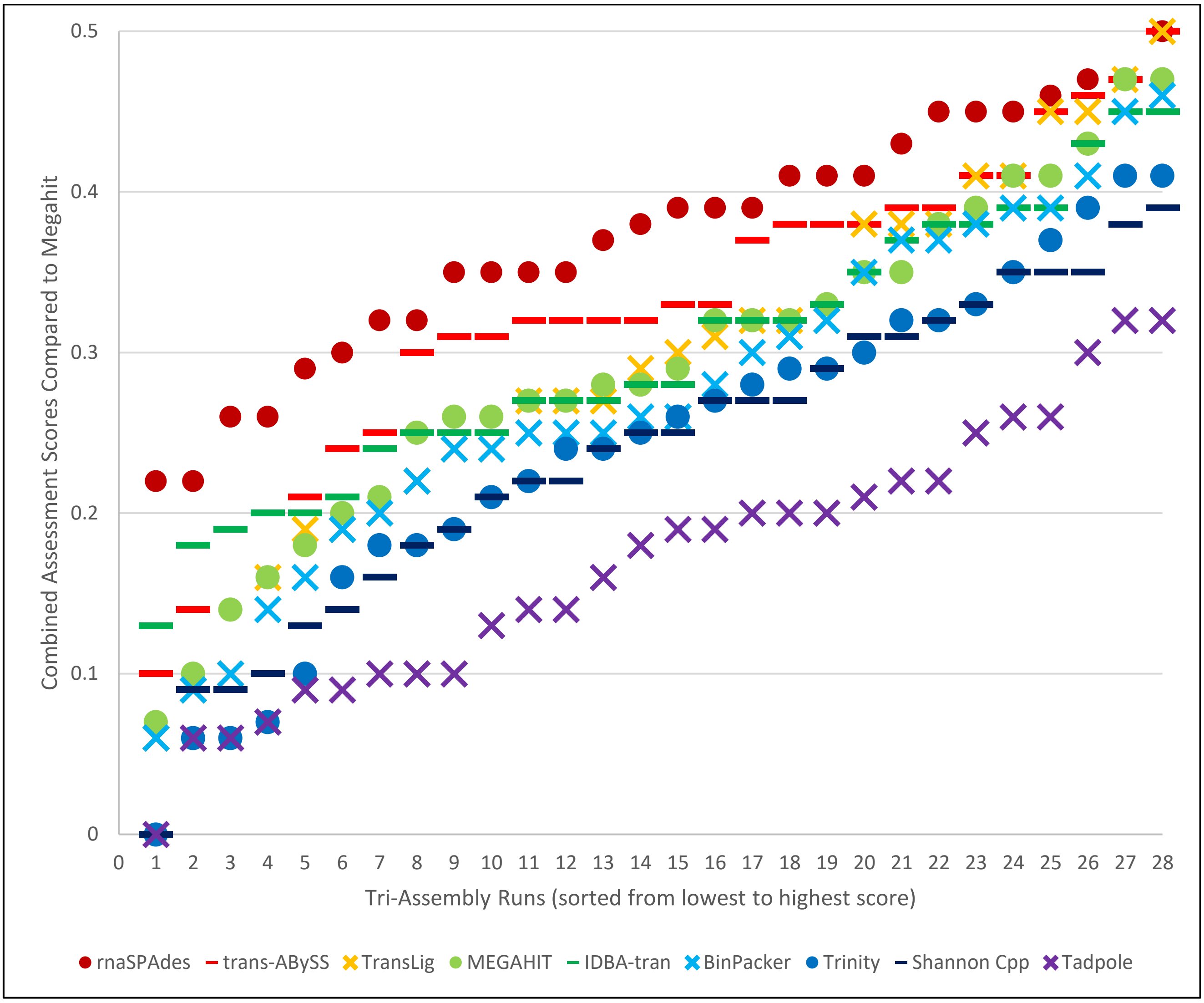

Figure S3. Tri-assembly runs sorted by lowest to highest assessment scores. All assembler metrics are compared to over/underperformance to the average megahit single-assembly score (0). By sorting from lowest to highest assessment scores per run we are able to depict the inherit variability involved in assembler combinations as well as a look into which assemblers are most consistent in groups of three.
